# Muscle function during single leg landing

**DOI:** 10.1038/s41598-022-15024-w

**Published:** 2022-07-07

**Authors:** Nirav Maniar, Anthony G. Schache, Claudio Pizzolato, David A. Opar

**Affiliations:** 1 School of Behavioural and Health Sciences, Australian Catholic University, Fitzroy, VIC Australia; 2 Sports Performance, Recovery, Injury and New Technologies (SPRINT) Research Centre, Australian Catholic University, Fitzroy, VIC Australia; 3 La Trobe Sport and Exercise Medicine Research Centre, La Trobe University, Bundoora, VIC Australia; 4 Griffith Centre of Biomedical and Rehabilitation Engineering, Menzies Health Institute Queensland, Griffith University, Gold Coast, Queensland Australia; 5 School of Health Sciences and Social Work, Griffith University, Gold Coast, Queensland Australia

**Keywords:** Musculoskeletal system, Mechanical engineering

## Abstract

Landing manoeuvres are an integral task for humans, especially in the context of sporting activities. Such tasks often involve landing on one leg which requires the coordination of multiple muscles in order to effectively dissipate kinetic energy. However, no prior studies have provided a detailed description of the strategy used by the major lower limb muscles to perform single-leg landing. The purpose of the present study was to understand how humans coordinate their lower limb muscles during a single-leg landing task. Marker trajectories, ground reaction forces (GRFs), and surface electromyography (EMG) data were collected from healthy male participants performing a single-leg landing from a height of 0.31 m. An EMG-informed neuromusculoskeletal modelling approach was used to generate neuromechanical simulations of the single-leg landing task. The muscular strategy was determined by computing the magnitude and temporal characteristics of musculotendon forces and energetics. Muscle function was determined by computing muscle contributions to lower limb net joint moments, GRFs and lower limb joint contact forces. It was found that the vasti, soleus, gluteus maximus and gluteus medius produced the greatest muscle forces and negative (eccentric) mechanical work. Downward momentum of the centre-of-mass was resisted primarily by the soleus, vasti, gastrocnemius, rectus femoris, and gluteus maximus, whilst forward momentum was primarily resisted by the quadriceps (vasti and rectus femoris). Flexion of the lower limb joints was primarily resisted by the uni-articular gluteus maximus (hip), vasti (knee) and soleus (ankle). Overall, our findings provide a unique insight into the muscular strategy used by humans during a landing manoeuvre and have implications for the design of athletic training programs.

## Introduction

Landing manoeuvres are an integral task for humans, particularly in the context of sporting activities. Many popular sports (e.g., football, basketball, etc.) require the ability to effectively land from various heights, often on one leg^1,2^. Importantly, these landing manoeuvres place substantial demands on the lower limbs in order to dissipate kinetic energy upon impact with the ground^3^. Failure to execute effective energy dissipation strategies during landing may place high mechanical demands on vulnerable tissues (e.g., joint structures such as ligaments, cartilage, etc.) potentially exposing them to mechanical damage and injury^4^.

Previous studies have attempted to quantify the biomechanical demands of landing tasks by measuring ground reaction forces and computing lower limb joint moments and powers^3–5^. These studies show that greater landing heights are associated with increases in some kinetic variables, particularly knee power absorption and vertical ground reaction forces^3,5^. Devita and Skelly^4^ showed that landing technique can redistribute joint work, with a “stiff” landing technique associated with greater ankle negative (eccentric) work and lower hip and knee negative work compared to a “soft” landing technique. However, this joint level analysis is limited since the human neuromuscular skeletal system is highly complex, consisting of hundreds of muscles with differing force and moment producing properties^6^. Importantly, each degree-of-freedom (DOF) is actuated by multiple muscles. Whilst this complexity permits the coordination of a wide range of challenging tasks (e.g., locomotion) with apparent relative ease^7^, it also complicates the determination of muscular strategies for a given task. One popular method to quantify muscular coordination is to use surface electromyography (EMG), which can be used to determine when specific muscles are active in a given task (e.g., see references^8–10^). However, these data do not establish a causal relationship between EMG patterns and biomechanical subtasks (e.g., supporting and propelling the body mass forwards during walking), thus no information is provided about the specific function of a muscle in a given movement^11^ (henceforth referred to as “muscle function”). Predicting muscle function is difficult as any muscle in the body can induce an acceleration of any segment in the body, due to a phenomenon known as “dynamic coupling”^12^.

Musculoskeletal modelling can account for dynamic coupling, and prior work has used modelling to gain insight into the coordination of human movement by determining how individual muscles contribute to biomechanical subtasks deemed fundamental to the overall movement pattern of interest. Depending on the nature of the activity, these studies have typically computed muscle force contributions to centre of mass accelerations, or equivalently, the ground reaction force (GRF), as well as joint moments, joint reaction loads and segmental power. Using such an approach, these studies have provided substantial insight into the muscular strategy used by humans in many fundamental movement patterns, such as walking^13–20^, running^21–23^, stair ambulation^24,25^, as well as gradual^26,27^ and sudden^28,29^ change of direction tasks. With respect to single-leg landing tasks, interpretations of muscle function have focused on how muscles induce loading of the anterior cruciate ligament^30,31^ (which is commonly injured during landing actions), neglecting analyses about how muscles coordinate landing itself. Importantly, muscle function is dependent on the kinematics of all segments in the system^12^, and is therefore expected to be task-specific.

Determining muscle function during landing provides the basis for any interventions aiming to improve overall landing performance. For example, determining how a muscle contributes to GRFs indicates how that muscle functions to arrest the centre-of-mass’s downward and forward momentum upon landing. Furthermore, collapse of the lower limb joints in the sagittal plane during landing requires flexion, thus determining how muscles contribute to the extension moments at each lower limb joint facilitates insight into an important component of landing. Finally, single-leg landing tasks are often investigated due to the large demands they place on lower limb joints (particularly the knee), thus determining how muscles contribute to joint loading may identify target muscles for any interventions aiming to reduce injury risk.

Subsequently, the purpose of this study was to investigate the muscular strategy used by the human lower limb during a single-leg landing task. Specifically, we used an EMG-informed neuromusculoskeletal modelling approach that accounted for participant-specific muscle recruitment patterns to estimate lower limb muscle function during landing. Our primary interest was to identify the magnitude and temporal characteristics of the individual lower limb muscle forces and mechanical energetics, as well as to determine each muscle’s contribution to the three fundamental biomechanical subtasks during landing, 1) to arrest the downward and forward momentum of the centre-of-mass, 2) to produce net joint moments at the lower limb and 3) to produce lower limb joint contact forces.

## Materials and methods

### Participants

A convenience sample of eight recreationally active healthy males (age: 27 ± 4 years; height: 1.77 ± 0.09 m; mass: 78 ± 13 kg) volunteered to participate in this study. All participants had no current or previous musculoskeletal injury likely to influence their ability to perform the required tasks. All participants provided written informed consent to participate in the study. Ethical approval was granted by the Australian Catholic University Human Research Ethics Committee (approval number: 2015-11H), and the study was carried out in accordance with the approved guidelines.

### Instrumentation

Three-dimensional marker trajectories were collected at 200 Hz using a nine-camera motion analysis system (VICON, Oxford Metrics Ltd., Oxford, United Kingdom). GRFs were collected via a ground-embedded force plate (Advanced Mechanical Technology Inc., Watertown, MA, USA) sampling at 1000 Hz. Surface EMG data were collected at 1000 Hz from 10 lower limb muscles on the dominant leg (defined as the kicking leg; right side for all participants) via two wireless EMG systems (Noraxon, Arizona, USA; Myon, Schwarzenberg, Switzerland) that were synchronised with the motion analysis system by accounting for each EMG system’s sensor delay.

### Procedures

All participants were barefoot during the experiment, which allowed exposure of the foot for marker placement. The skin was prepared for surface EMG collection by shaving, abrasion and sterilisation. Circular bipolar pre-gelled Ag/AgCl electrodes (inter-electrode distance of 2 cm) were then placed on the vastus lateralis and medialis, rectus femoris, biceps femoris, medial hamstrings (semimembranosus and semitendinosus), medial and lateral gastrocnemius, soleus, tibialis anterior and peroneus longus muscles in accordance with Surface Electromyography for the Non-Invasive Assessment of Muscle (SENIAM) guidelines^32^. EMG-time traces during forceful isometric contractions were visually inspected to verify the correct placement of the electrodes and to evaluate for evidence of cross-talk. Additionally, participants were required to perform isometric maximum voluntary contraction trials (knee flexion and extension, ankle plantar and dorsi-flexion) in order to normalise the EMG data^31^. After completion of the maximum voluntary contractions, 43 retroreflective markers (14 mm) were affixed to various anatomical locations on the whole body as previously described^28,29^.

Each participant completed a single-leg landing task on their right leg. Prior to recording data, participants performed bilateral drop jump and single-leg landing tasks in order to prepare and familiarise themselves with the experimental procedures. Participants were then required to perform a single-leg landing on their right leg after dropping off a box (height = 0.31 m). Participants were required to land with their entire foot within the boundaries of the ground embedded force plate (positioned immediately anterior to the box) and, without shuffling or sliding their feet, rise from the point of peak knee flexion to an upright pose (with a fully extended knee) without any other part of their body (e.g., their contralateral foot) touching the ground at any point. Participants were informed of the criteria for a successful trial before performing the task, but no specific technique coaching was provided prior to or during testing. A single successful trial from each participant was selected for subsequent analysis.

### Data processing

Marker trajectories and GRFs were low-pass filtered using a zero-lag, 4th order Butterworth filter with a cut-off frequency of 15 Hz. EMG data were corrected for offset, high pass filtered (20 Hz), full-wave rectified and low-pass filtered (6 Hz) using a zero-lag, 4th order Butterworth filter to obtain a linear envelope. EMG data were normalised to the peak amplitude obtained across all reference trials. These reference trials included both the isometric maximum voluntary contractions as well as the dynamic tasks.

### Musculoskeletal modelling

A 31 DOF full-body musculoskeletal model, with 80 musculotendon actuators (lower body) and 19 force/torque actuators (upper body)^33^, was used to perform the musculoskeletal simulations in OpenSim^34^. Each hip was modelled as a 3-DOF ball and socket. Each knee was modelled as a 1-DOF hinge, with other rotational (valgus/varus and internal/external rotation) and translational (anteroposterior and superior-inferior) movements constrained to change as a function of the knee flexion angle^35^. Two non-intersecting pin joints were used to represent the ankle (talocrural and subtalar joints). The head-trunk segment was modelled as a single rigid segment, articulating with the pelvis via a 3-DOF ball and socket joint. Each upper limb was characterised by a 3-DOF ball and socket shoulder joint and single-DOF elbow and radioulnar joints. The generic model was linearly scaled to each participant’s individual anthropometry as determined during a static trial. An inverse kinematics algorithm was used to calculate joint angles by means of a least-squares optimisation that minimised the difference between model and experimental marker positions^36^. Inverse dynamics was used to obtain the joint moments acting about each modelled DOF. We then computed muscle–tendon unit lengths and moment arms about the respective joints they crossed.

Musculotendon forces were obtained via an EMG-assisted optimisation algorithm^37,38^. To perform these simulations, the inverse dynamics derived joint moments were combined with the computed musculotendon lengths, muscle moment arms, and the normalised EMG signal to calibrate muscle–tendon unit parameters in the scaled musculoskeletal model^37^. This process utilised a simulated annealing algorithm to minimise the difference between experimental joint moments (computed from inverse dynamics) and model joint moments (computed as the product of muscle forces and their corresponding moment arms) by adjusting neuromuscular skeletal parameters (optimal fibre length, tendon slack lengths) within uncertainty tolerances. After this calibration process, an EMG-assisted algorithm estimated muscle forces by using a static optimisation algorithm to decompose net joint moments into individual muscle forces by minimising the sum of activations squared, whilst also limiting the deviation of the excitation patterns from experimentally recorded EMG (for muscles where EMG data were recorded). This method therefore accounted for participant-specific muscle recruitment patterns for muscles which had EMG data available, and constrained the solution space for remaining muscles actuating the same degrees of freedom. Note that since EMG data were only recorded from the stance leg, the musculotendon forces of the contralateral leg were solved using static optimisation. Musculotendon forces and their contributions to the net joint moments were extracted for further analysis. Due to the impulse-momentum relationship, contributions to the net joint moment were also integrated with respect to time, in order to compute the net angular impulse produced by each muscle to arrest ‘collapse’ of lower limb joints (e.g., flexion in the sagittal plane) during landing. Additionally, mechanical power developed by each muscle was computed by multiplying musculotendon force by its respective musculotendinous velocity. Mechanical work was also computed by integrating the muscle power-time curve. For both mechanical power and work, negative values indicate power/energy absorption (eccentric contraction), whilst positive values represent power/energy generation (concentric contraction).

The measured GRFs were decomposed into individual muscular contributions by using a universal “rolling on ground” constraint to model the interaction between the foot and the ground^21,39^. Since the task was a drop landing, we focused on each muscle’s contribution to the vertical GRF, as this represents each muscle’s contribution to opposing the downward pull of gravity during the landing task. Additionally, since the platform was positioned directly behind the force plate, an appreciable forward motion was required in order for the participant to land on the force plate, thus we also computed contributions to the anteroposterior GRF. Due to the impulse-momentum relationship, contributions to the GRF were integrated with respect to time, in order to compute the net impulse produced by each muscle to arrest the downward and anterior momentum of the body during landing.

Each muscle’s contribution to lower limb joint loading was determined by computing muscle contributions to the hip, knee and ankle compressive contact force. These values were computed by applying each muscle’s force and contribution to the GRF in isolation and resolving the dynamical equations of motion and computing the contact force expressed in the reference frame of the distal body segment. These contact forces were also integrated with respect to time to determine their “net” effect whilst landing^40^.

### Outcome variables

Musculotendon force, mechanical power, mechanical work, as well as the contributions to net joint moments, GRFs and contact forces were extracted from the landing phase (defined as the time-period from initial foot contact to peak knee flexion). All force related data (i.e., musculotendon force, GRFs and contact forces) were normalised to bodyweight (BW), whilst all other variables (mechanical power/work and joint moments) were normalised to body mass. Each discrete outcome variable was then averaged across participants and plotted for interpretation. For time-varying quantities, data were first time-normalised to 101 points (0–100% of landing phase), and ensemble averages were then plotted for interpretation. Musculotendon outcome variables were grouped according to functional groups: ADDLONG (adductor brevis and longus), ADDMAG (adductor magnus), BFSH (biceps femoris short head), DORSI (tibialis anterior, extensor digitorum longus, extensor hallucis longus), GAS (medial and lateral gastrocnemius), GMAX (gluteus maximus), GMED (gluteus medius and gluteus minimus), HAM (biceps femoris long head, semimembranosus, semitendinosus), ILPSO (iliacus and psoas major), PER (peroneus brevis and longus), PFINV (plantar-flexor-invertors: tibialis posterior, flexor digitorum longus, flexor hallucis longus), PIRI (piriformis), RECFEM (rectus femoris), SOLEUS (soleus), VASTI (vastus medialis, lateralis and intermedius).

### Validation and verification

To provide confidence in our simulations, we performed validation and verification according to best practice recommendations^41^. Similar time-varying characteristics of our experimental joint angles^31^ and inverse dynamics based joint moments (Supplementary Fig. S1 online) were observed when compared with prior published data of single-leg landing tasks from similar heights^3,30,42^. We also verified a good match between experimental and simulated (from the EMG-assisted model) variables, including our predicted joint moments and inverse dynamics derived joint moments (Supplementary Fig. S1 online), predicted excitations and experimental EMG data (Supplementary Fig. S2 online), experimentally measured GRFs and simulated GRFs (Supplementary Fig. S3 online), as well as between the joint contact forces derived from experimental and simulated GRFs (Supplementary Fig. S4 online).

## Results

### Muscle forces, mechanical work and power

**Figure 1:**
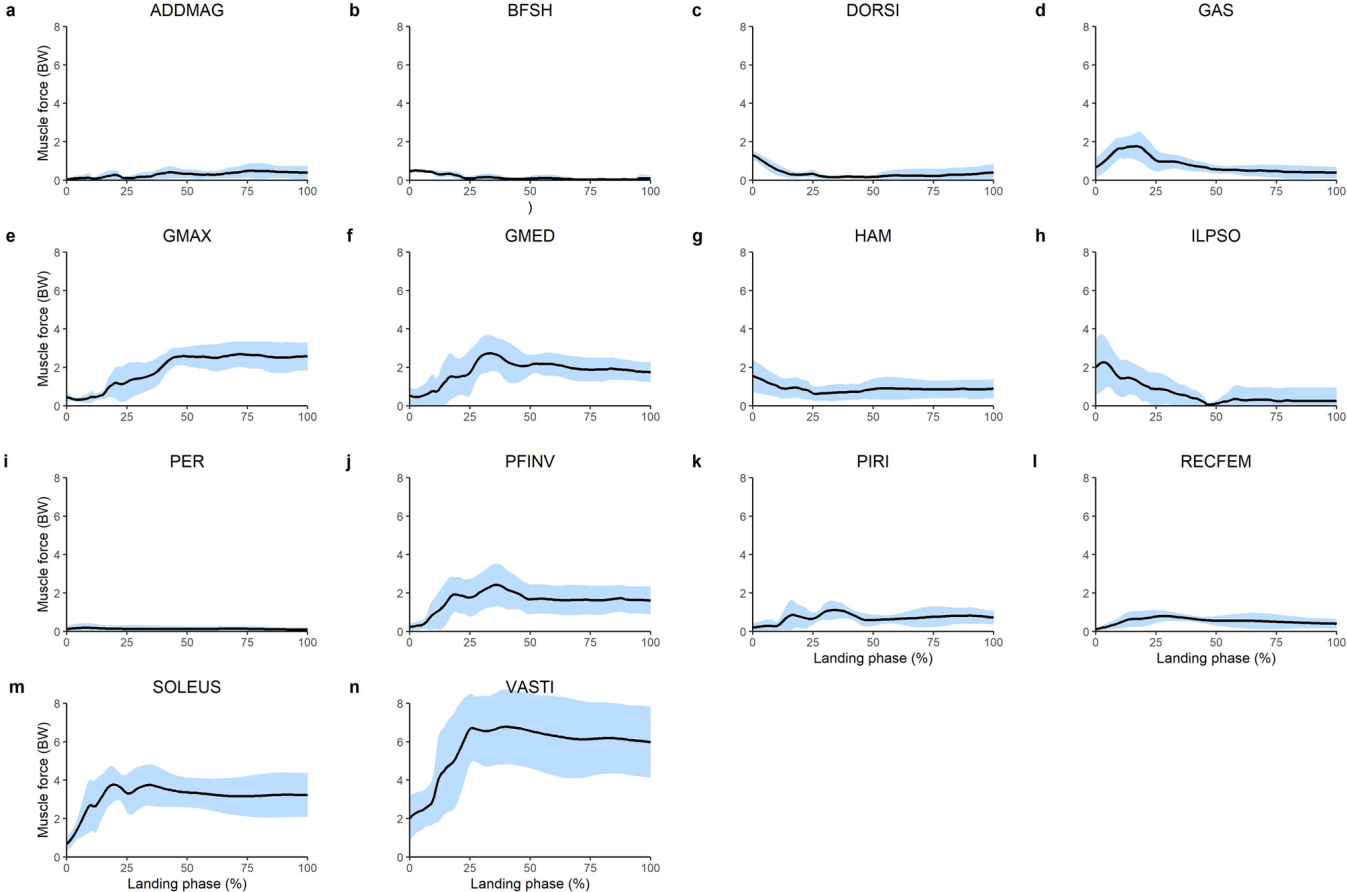
Mean (black line) and SD (blue shaded) bodyweight (BW) normalised lower limb musculotendinous forces for the landing phase (initial contact to peak knee flexion) of a single-leg drop landing from a 0.31 m height. ADDMAG, adductor magnus; BFSH, biceps femoris short head; DORSI, ankle dorsiflexors (sum of tibialis anterior, extensor hallucis and digitorum longus); GAS, gastrocnemius (sum of lateral and medial gastrocnemius); GMAX, gluteus maximus; GMED, gluteus medius (sum of gluteus medius and minimius), HAM, biarticular hamstrings (sum of biceps femoris long head, semimembranosus, and semitendinosus); ILPSO, iliopsoas (sum of iliacus and psoas major); PER, peroneus (sum of peroneus longus and brevis); PFINV, plantar flexor invertors (sum of tibialis posterior, flexor hallucis and digitorum longus); PIRI, piriformis; RECFEM, rectus femoris; SOLEUS, soleus; VASTI, vasti (sum of vastus intermedius, lateralis and medialis).

**Figure 2:**
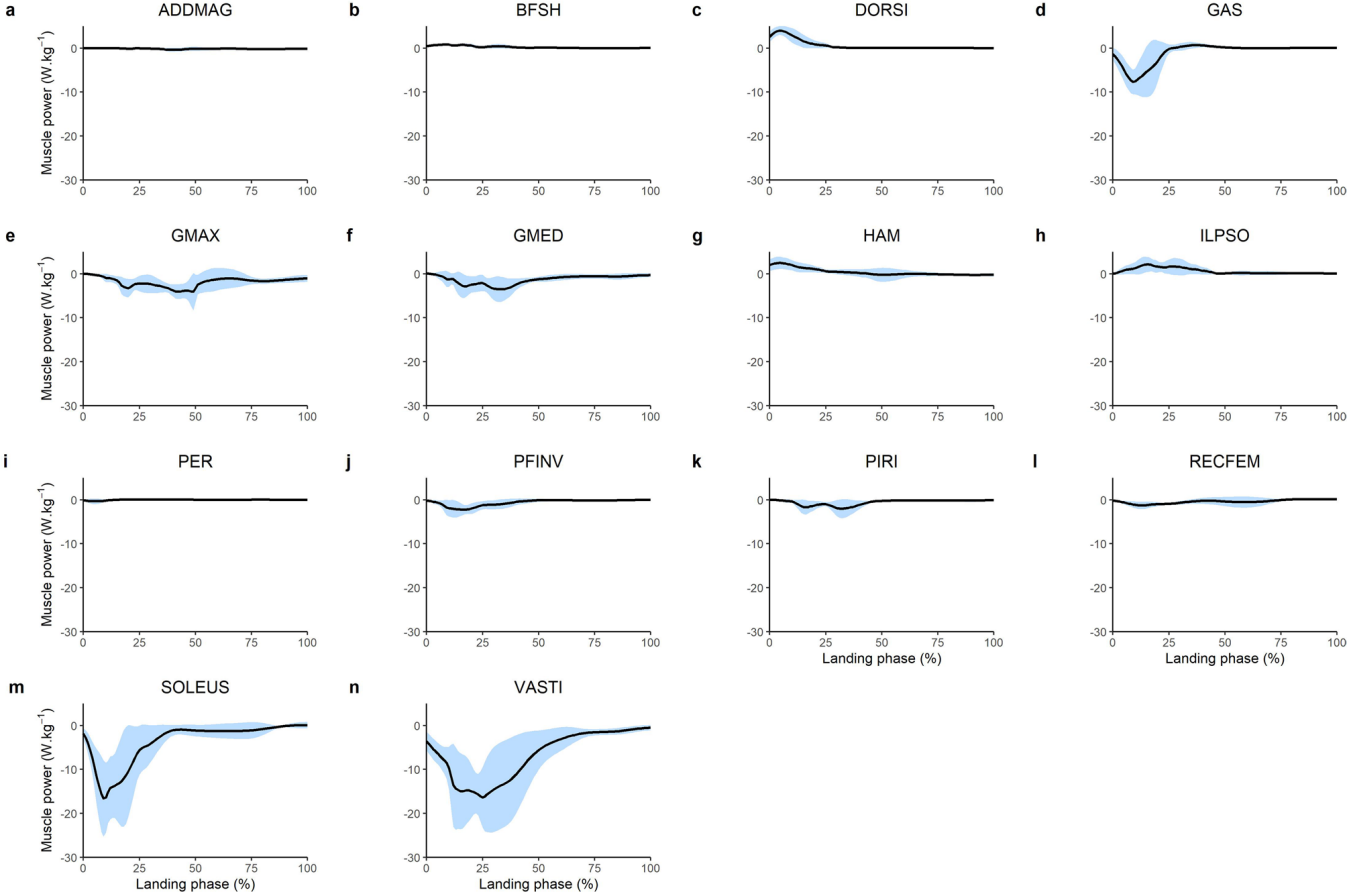
Mean (black line) and SD (blue shaded) body mass normalised lower limb musculotendinous mechanical power for the landing phase (initial contact to peak knee flexion) of a single-leg drop landing from a 0.31 m height. ADDMAG, adductor magnus; BFSH, biceps femoris short head; DORSI, ankle dorsiflexors (sum of tibialis anterior, extensor hallucis and digitorum longus); GAS, gastrocnemius (sum of lateral and medial gastrocnemius); GMAX, gluteus maximus; GMED, gluteus medius (sum of gluteus medius and minimius), HAM, biarticular hamstrings (sum of biceps femoris long head, semimembranosus, and semitendinosus); ILPSO, iliopsoas (sum of iliacus and psoas major); PER, peroneus (sum of peroneus longus and brevis); PFINV, plantar flexor invertors (sum of tibialis posterior, flexor hallucis and digitorum longus); PIRI, piriformis; RECFEM, rectus femoris; SOLEUS, soleus; VASTI, vasti (sum of vastus intermedius, lateralis and medialis).

**Figure 3:**
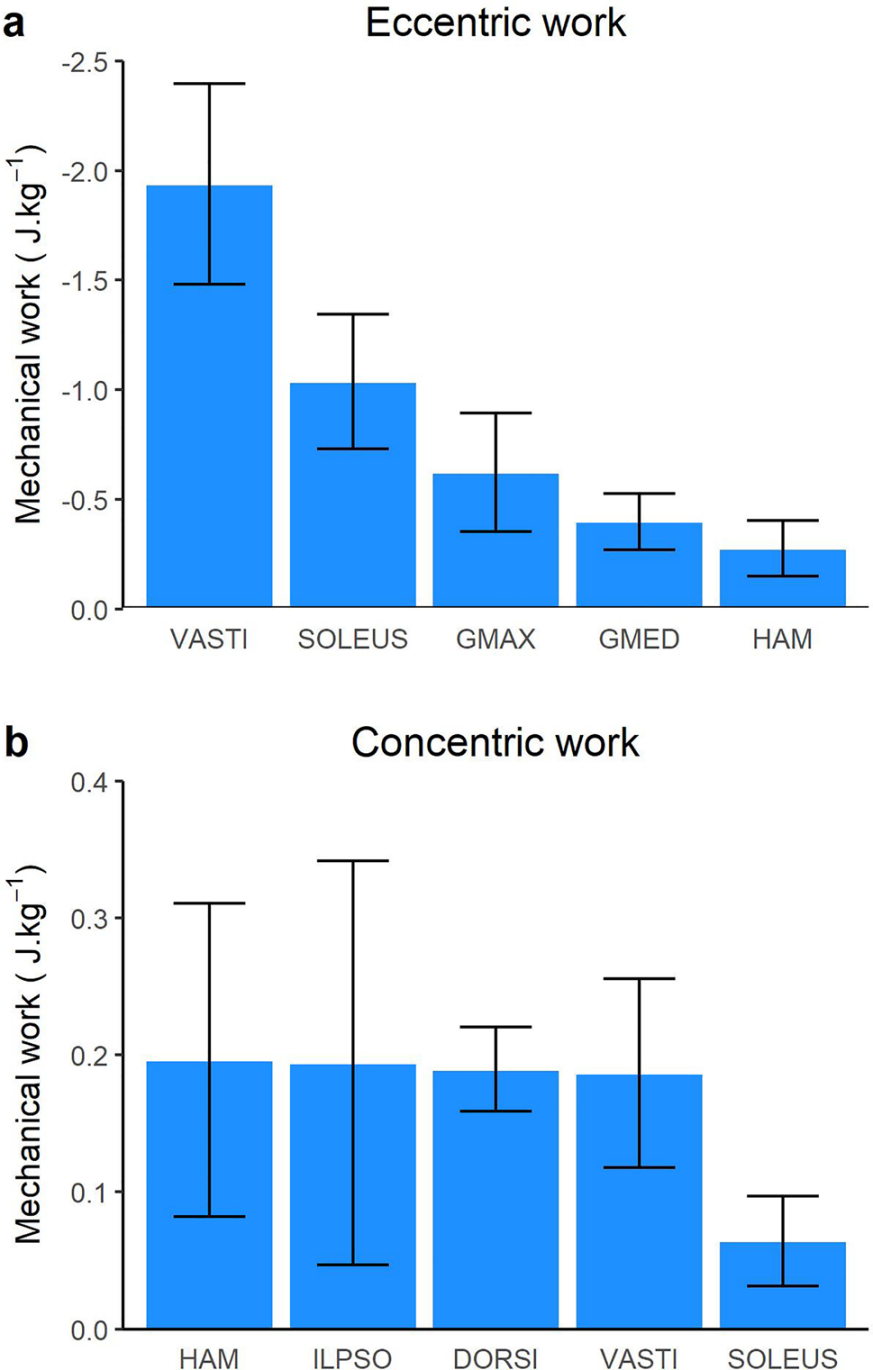
Mean (bars) and SD (error bars) body mass normalised lower limb musculotendinous eccentric (**a**) and concentric (**b**) mechanical work across the landing phase (initial contact to peak knee flexion) of a single-leg drop landing from a 0.31 m height. VASTI, vasti (sum of vastus intermedius, lateralis and medialis); SOLEUS, soleus; GMAX, gluteus maximus; GMED, gluteus medius (sum of gluteus medius and minimius), HAM, biarticular hamstrings (sum of biceps femoris long head, semimembranosus, and semitendinosus); ILPSO, iliopsoas (sum of iliacus and psoas major); DORSI, ankle dorsiflexors (sum of tibialis anterior, extensor hallucis and digitorum longus).

The greatest muscle forces were produced by the uni-articular lower limb joint extensors (Fig. 1). The VASTI produced the greatest peak force (6.77 BW) followed by the SOLEUS (3.77 BW), GMED (2.74 BW) and the GMAX (2.68 BW). These muscles, along with the majority of the lower limb muscles, absorbed power (i.e. performed negative/eccentric mechanical work) throughout the landing phase (Fig. 2). The greatest amount of eccentric mechanical work was performed by the VASTI, SOLEUS and GMAX. These muscles produced 1.94 J kg^−1^, 1.04 J kg^−1^ and 0.62 J kg^−1^ of eccentric work, respectively (Fig. 3). Eccentric work was done by these muscles throughout the entire landing phase, although most of this eccentric work occurred within the first 40–60% of the landing phase (Fig. 2). The GMED, GAS and HAMS produced 0.40 J kg^−1^, 0.35 J kg^−1^and 0.28 J kg^−1^ of eccentric work, respectively. The GMED and GAS performed most of this eccentric work during the first half of the landing phase, whilst the HAMS typically performed eccentric work during the mid-portion (40–50%) of the landing phase (Fig. 2). All other muscles contributed less than 0.2 J kg^−1^ of eccentric work each. The greatest amount of positive work was typically produced by muscles that contributed to flexion moments about the lower limb joints, including the knee flexing HAMS (0.20 J kg^−1^), hip-flexing ILPSO (0.19 J kg^−1^), and ankle flexing DORSI (0.19 J kg^−1^). Most of this concentric work was performed during the first 30–40% of the landing phase (Fig. 2).

### Contributions to GRF

**Figure 4:**
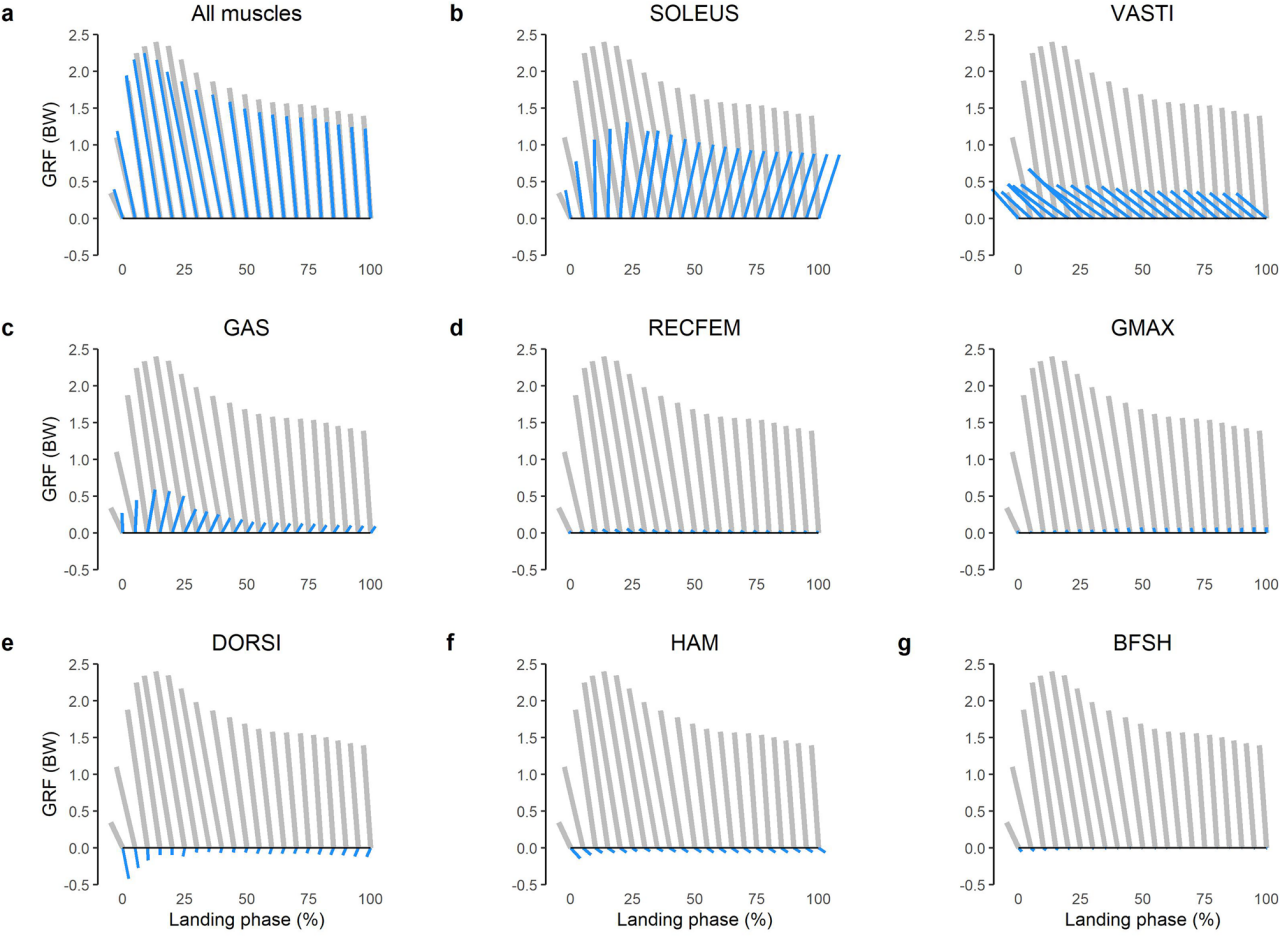
Mean musculotendinous contributions to the bodyweight (BW) normalised ground reaction force (GRF) for the landing phase (initial contact to peak knee flexion) of a single-leg drop landing from a 0.31 m height. Vectors oriented upward and to the right indicate superiorly and anteriorly directed GRFs, respectively. Grey vectors represent the net GRF measured from ground embedded force plate, whilst blue vector indicates muscle contributions as follows: All muscles, sum of all musculotendinous actuators in the model; SOLEUS, soleus; VASTI, vasti (sum of vastus intermedius, lateralis and medialis); GAS, gastrocnemius (sum of lateral and medial gastrocnemius) RECFEM, rectus femoris; GMAX, gluteus maximus; DORSI, ankle dorsiflexors (sum of tibialis anterior, extensor hallucis and digitorum longus); HAM, biarticular hamstrings (sum of biceps femoris long head, semimembranosus, and semitendinosus); BFSH, biceps femoris short head.

**Figure 5:**
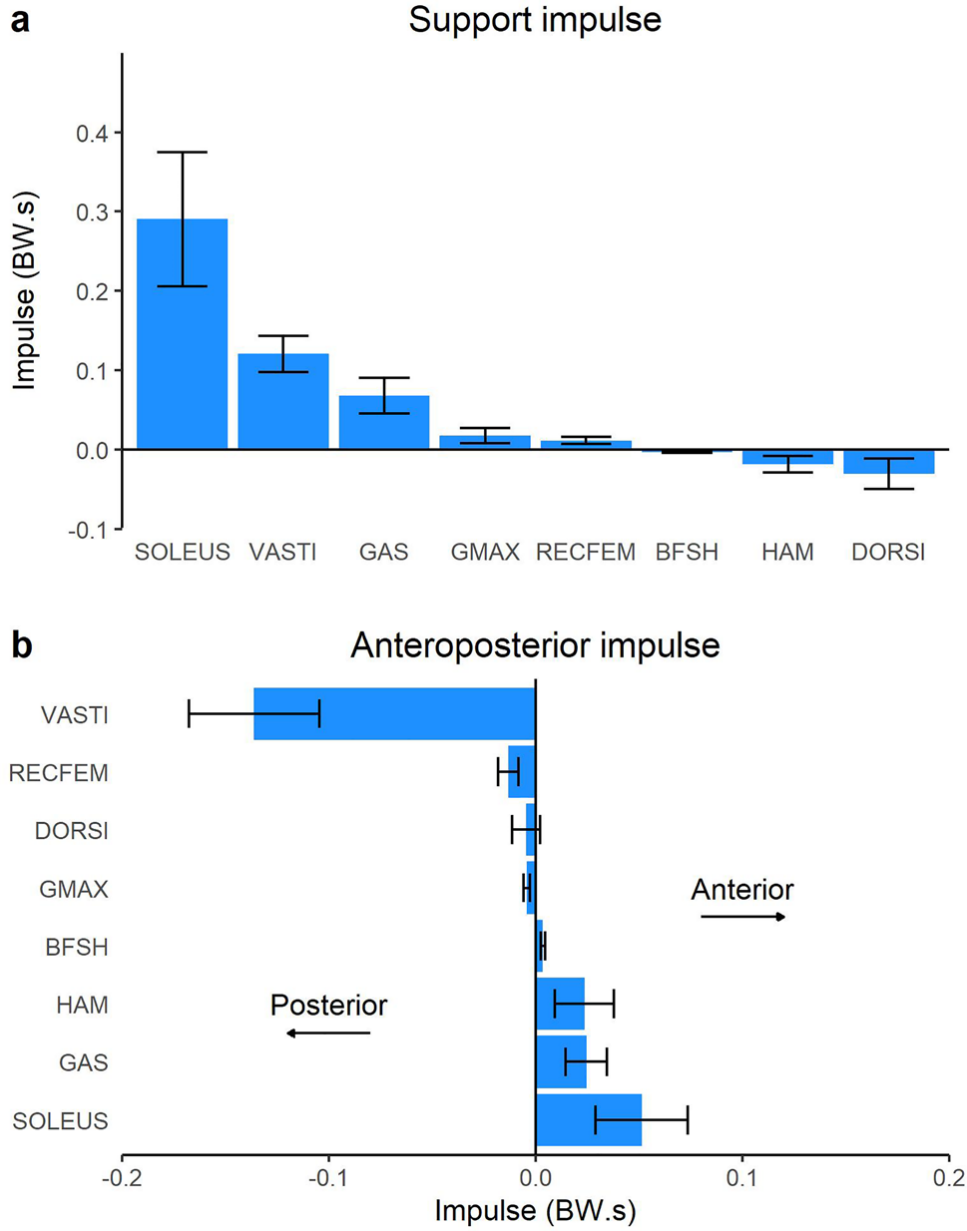
Mean (bars) and SD (error bars) musculotendinous contributions to the bodyweight (BW) normalised ground reaction force impulse across the landing phase (initial contact to peak knee flexion) of a single-leg drop landing from a 0.31 m height. SOLEUS, soleus; VASTI, vasti (sum of vastus intermedius, lateralis and medialis); GAS, gastrocnemius (sum of lateral and medial gastrocnemius) GMAX, gluteus maximus; RECFEM, rectus femoris; BFSH, biceps femoris short head; HAM, biarticular hamstrings (sum of biceps femoris long head, semimembranosus, and semitendinosus); DORSI, ankle dorsiflexors (sum of tibialis anterior, extensor hallucis and digitorum longus).

The net GRF was directed upwards and posteriorly throughout the landing phase (Fig. 4). The GRF was primarily modulated by the lower limb extensor muscles, with the ankle plantar flexors (SOLEUS and GAS) generating GRFs directed upwards and anteriorly, and the quadriceps (VASTI and RECFEM) and GMAX generating GRFs directed upwards and posteriorly (Fig. 4). An upwards (vertical support) impulse was primarily provided by the SOLEUS, producing 0.29 BW s across the landing phase (Fig. 5a), with secondary contributions from the VASTI (0.12 BW s), GAS (0.07 BW s), GMAX (0.02 BW s) and RECFEM (0.01 BW s). These same 5 muscles, along with HAM, were primarily responsible for modulating the GRF in the anteroposterior direction. A posteriorly directed (braking) impulse was primarily produced by VASTI (0.14 BW s) and RECFEM (0.01 BW s), whilst an anteriorly directed impulse was primarily produced by SOLEUS (0.05 BW s), GAS (0.02 BW s) and HAM (0.02 BW s) (Fig. 5b).

### Contributions to joint moments

**Figure 6:**
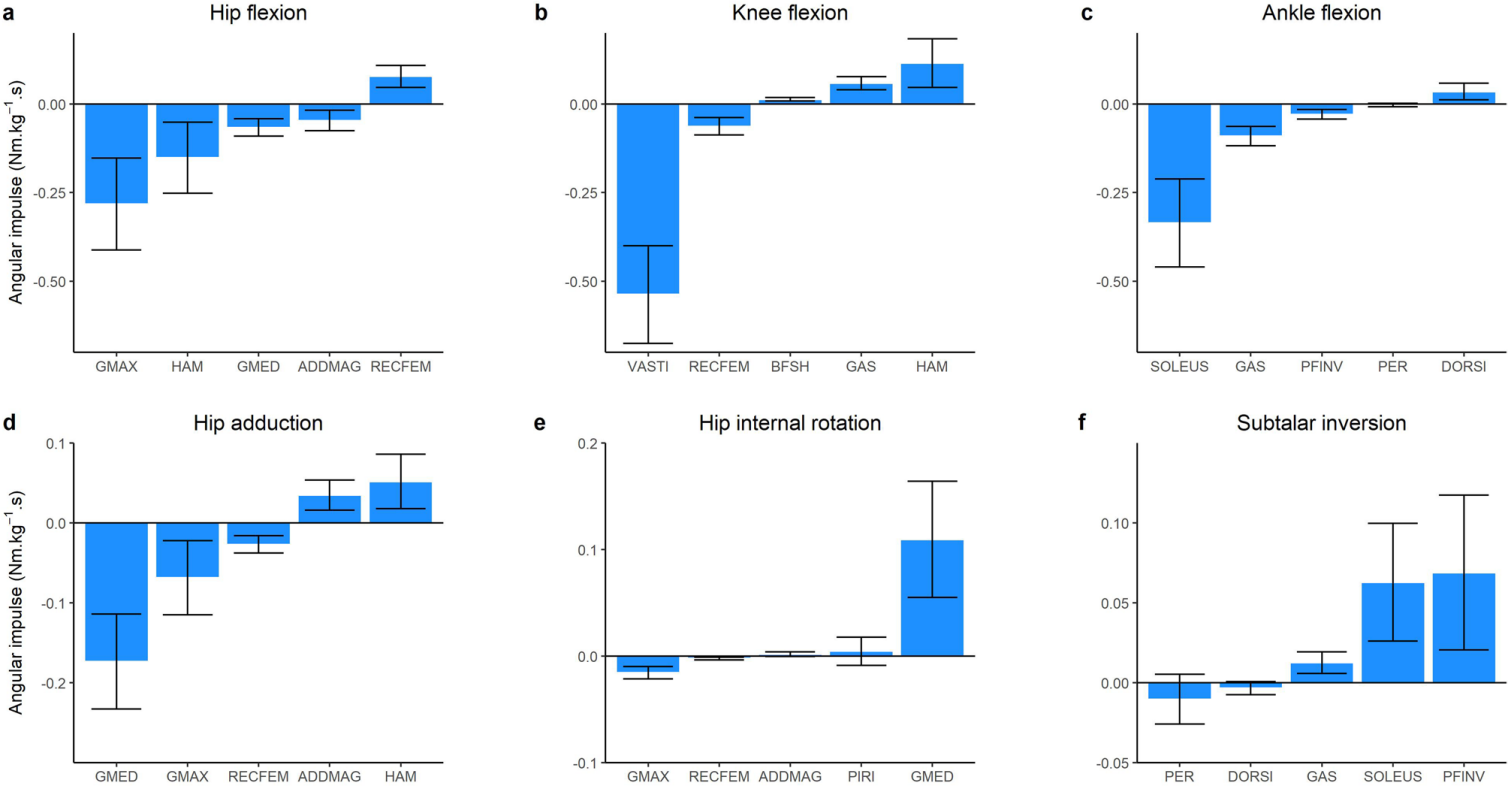
Mean (bars) and SD (error bars) musculotendinous contributions to the body mass normalised joint angular impulse across the landing phase (initial contact to peak knee flexion) of a single-leg drop landing from a 0.31 m height. Positive values are indicated by the y-axis title. GMAX, gluteus maximus; HAM, biarticular hamstrings (sum of biceps femoris long head, semimembranosus, and semitendinosus); GMED, gluteus medius (sum of gluteus medius and minimius), ADDMAG, adductor magnus; RECFEM, rectus femoris; VASTI, vasti (sum of vastus intermedius, lateralis and medialis); BFSH, biceps femoris short head; GAS, gastrocnemius (sum of lateral and medial gastrocnemius); SOLEUS, soleus; PFINV, plantar flexor invertors (sum of tibialis posterior, flexor hallucis and digitorum longus); PER, peroneus (sum of peroneus longus and brevis); DORSI, ankle dorsiflexors (sum of tibialis anterior, extensor hallucis and digitorum longus); PIRI, piriformis.

In the sagittal plane, each lower limb joint experienced an external flexion moment throughout the entire landing phase, which was countered by an internal extension angular impulse of 0.42 Nm kg^−1^ s, 0.44 Nm kg^−1^ s, and 0.47 Nm kg^−1^ s for the hip, knee and ankle, respectively. These impulses were primarily generated by uni-articular muscles, i.e. GMAX at the hip (0.28 Nm kg^−1^ s), VASTI at the knee (0.54 Nm kg^−1^ s) and SOLEUS at the ankle (0.34 Nm kg^−1^ s) (Fig. 6a–c). Bi-articular muscles also contributed to the internal extension angular impulses (albeit of a smaller magnitude), including the HAM at the hip (0.15 Nm kg^−1^ s), RECFEM at the knee (0.06 Nm kg^−1^ s) and GAS at the ankle (0.09 Nm kg^−1^ s). Opposing contributions (i.e. flexion moments) were primarily generated by the RECFEM at the hip (0.08 Nm kg^−1^ s), HAM at the knee (0.12 Nm kg^−1^ s), and DORSI at the ankle (0.03 Nm kg^−1^ s).

In the frontal plane, net abduction and inversion moments were observed throughout the landing phase, peaking at 0.24 Nm kg^−1^ and 0.13 Nm kg^−1^, respectively. Contributions to the hip abduction angular impulse primarily came from GMED (0.17 Nm kg^−1^ s) and GMAX (0.07 Nm kg^−1^ s), with antagonist (adduction) contributions from HAM (0.05 Nm kg^−1^ s) and ADDMAG (0.03 Nm kg^−1^ s) (Fig. 6d, e). At the subtalar joint, the net inversion moment was primarily produced by PFINV (0.07 Nm kg^−1^ s) and SOLEUS (0.06 Nm kg^−1^ s), whereas PER and DORSI produced small internal eversion angular impulses of no more than 0.01 Nm kg^−1^ s.

### Contributions to joint contact loading

**Figure 7:**
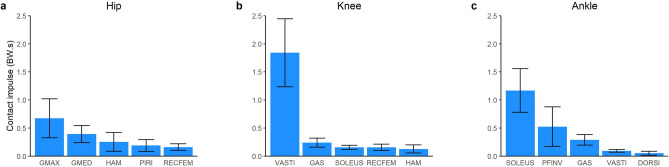
Mean (bars) and SD (error bars) musculotendinous contributions to the bodyweight (BW) normalised joint contact impulse across the landing phase (initial contact to peak knee flexion) of a single-leg drop landing from a 0.31 m height. GMAX, gluteus maximus; GMED, gluteus medius (sum of gluteus medius and minimius); HAM, biarticular hamstrings (sum of biceps femoris long head, semimembranosus, and semitendinosus); PIRI, piriformis; RECFEM, rectus femoris; VASTI, vasti (sum of vastus intermedius, lateralis and medialis); GAS, gastrocnemius (sum of lateral and medial gastrocnemius); SOLEUS, soleus; PFINV, plantar flexor invertors (sum of tibialis posterior, flexor hallucis and digitorum longus); DORSI, ankle dorsiflexors (sum of tibialis anterior, extensor hallucis and digitorum longus).

Throughout the landing phase, the hip, knee and ankle joints experienced a net compressive contact impulse of 2.20 BW s, 2.72 BW s and 2.22 BW s, respectively. The total hip contact impulse was primarily produced by GMAX (0.67 BW s) and GMED (0.39 BW s), with secondary contributions from HAM (0.25 BW s), PIRI (0.19 BW s) and RECFEM (0.16 BW s) (Fig. 7). The quadriceps made the largest contribution to the knee contact impulse (VASTI, 1.84 BW s; RECFEM, 0.15 BW s), with secondary contributions from GAS (0.24 BW s), SOLEUS (0.15 BW s), HAM (0.13 BW s). SOLEUS made the largest contribution to the ankle joint contact impulse across the landing phase (1.17 BW s) followed by the PFINV (0.52 BW s), GAS (0.29 BW s) and VASTI (0.09 BW s).

## Discussion

This study has described the muscular strategy employed by humans during single-leg drop landings. Notably, we found that the uni-articular lower limb joint extensors (VASTI, SOLEUS, GMAX and GMED) produced the greatest peak muscle forces and performed the greatest amount of eccentric work. The VASTI and SOLEUS, along with the bi-articular GAS, provided the greatest contributions to vertical support throughout the landing phase. These muscles also modulated anteroposterior centre-of-mass momentum, with SOLEUS and GAS producing an anteriorly directed impulse and VASTI and RECFEM producing a posteriorly directed impulse. Moreover, VASTI, SOLEUS and GMAX prevented lower limb joint flexion (by contributing to joint extension moments), whilst other muscles were important for preventing hip adduction (GMED) and subtalar eversion (PFINV). Both uni-articular and bi-articular (e.g., HAMS, RECFEM and GAS) muscles contributed substantially to lower limb joint contact forces, including to joints that they do not cross in some cases (e.g., SOLEUS at the knee). To the authors’ knowledge, no previous studies have used musculoskeletal modelling to provide a complete understanding of lower limb muscle function during single-leg drop landings.

### Muscle forces and mechanical work

Our estimates of muscle function require muscle force estimation, thus validation of muscle forces is an important first step to interpret our findings. Whilst direct validation of our estimated muscle forces is not feasible, the validity of the muscle forces in the present work is supported by two previous studies^30,42^ that have reported muscle forces during a 0.3 m single-leg drop landing. For example, the peak SOLEUS and HAM muscle forces of 3.77 BW (2836 N) and 1.55 BW (1144 N), respectively, are similar to the ~ 3500 N and ~ 1000 N reported by Mokhtarzadeh and colleagues^30^. In contrast, this same study reported peak quadriceps forces of ~ 2225 N, which is substantially less than the 6.77 BW (5075 N) observed in the present study. This is likely due differences in the landing tasks, as participants in the previous study^30^ were instructed to hop forward after landing, thus their strategy during the landing phase may have been different to that used by participants in the current study. Cleather and Czasche^42^ had participants perform a task more consistent with the present study and reported peak quadriceps forces of ~ 4 to 6 BW which approximates our own estimates. Additionally, their predicted muscle forces for the triceps surae (i.e., sum of GAS and SOLEUS, ~ 4 to 6 BW) and gluteals (i.e., sum of GMAX and GMED, ~ 2 to 4 BW) were consistent with our computations of 5.47 BW and 4.75 BW, respectively.

The majority of the lower limb muscles performed negative work throughout the landing phase (Fig. 2). This observation is similar to the landing phase of running^43^ and sidestep cutting^28^. Whilst we are unaware of other studies reporting muscle mechanical work and power during single-leg drop landing, prior work has determined the net knee joint power absorption during single-leg landing^5^ and found a similar temporal pattern to our observations for the VASTI (a uni-articular knee extensor, Fig. 2b), which provides some indirect validation of our findings. Intuitively, the substantial negative work performed by the lower limb joint extensors is unsurprising given their observed role in decelerating the centre-of-mass (Figs. 4, 5) and preventing lower limb joint flexion (Fig. 6).

### Arresting centre of mass momentum

The major contributors to an upwards impulse (vertical support) reported in the present work (SOLEUS, VASTI, GAS, RECFEM, GMAX) are similar to data reported in prior investigations of locomotion tasks, including walking^7,17–20^, running^21^, sidestep cutting^28^ and stair ambulation^24^. Additionally, the key muscles for generating a posteriorly directed impulse (braking) (VASTI, RECFEM, DORSI and GMAX) were also similar to these prior studies. However, some discrepancies between the present data and prior work are evident. For example, the relative contribution of the GMED to vertical support is lower in the present work compared to prior work investigating walking^7,17,18^. This may be because the contribution of a muscle to the GRF is dependent not only on the magnitude of the muscle forces, but the kinematics of all segments in the system^12^. Therefore, the differing kinematics between landing and previously investigated tasks may also explain differing muscle contributions and highlights the importance of task-specific investigations.

### Contribution to sagittal plane net joint moments

As collapse of the lower limb joints in the sagittal plane involves flexion, contributions to the extension moments at each lower limb joint are an important component of supporting the body during landing. The present work suggests that the hip, knee and ankle extension moments are largely produced by the uni-articular GMAX, VASTI and SOLEUS, respectively, with secondary contributions from the bi-articular HAM (at the hip), RECFEM (at the knee) and GAS (at the ankle) (Fig. 3a). This observation is similar to prior investigations of other high impact tasks, including running^23^ and sidestep cutting^28^.

**Figure 8:**
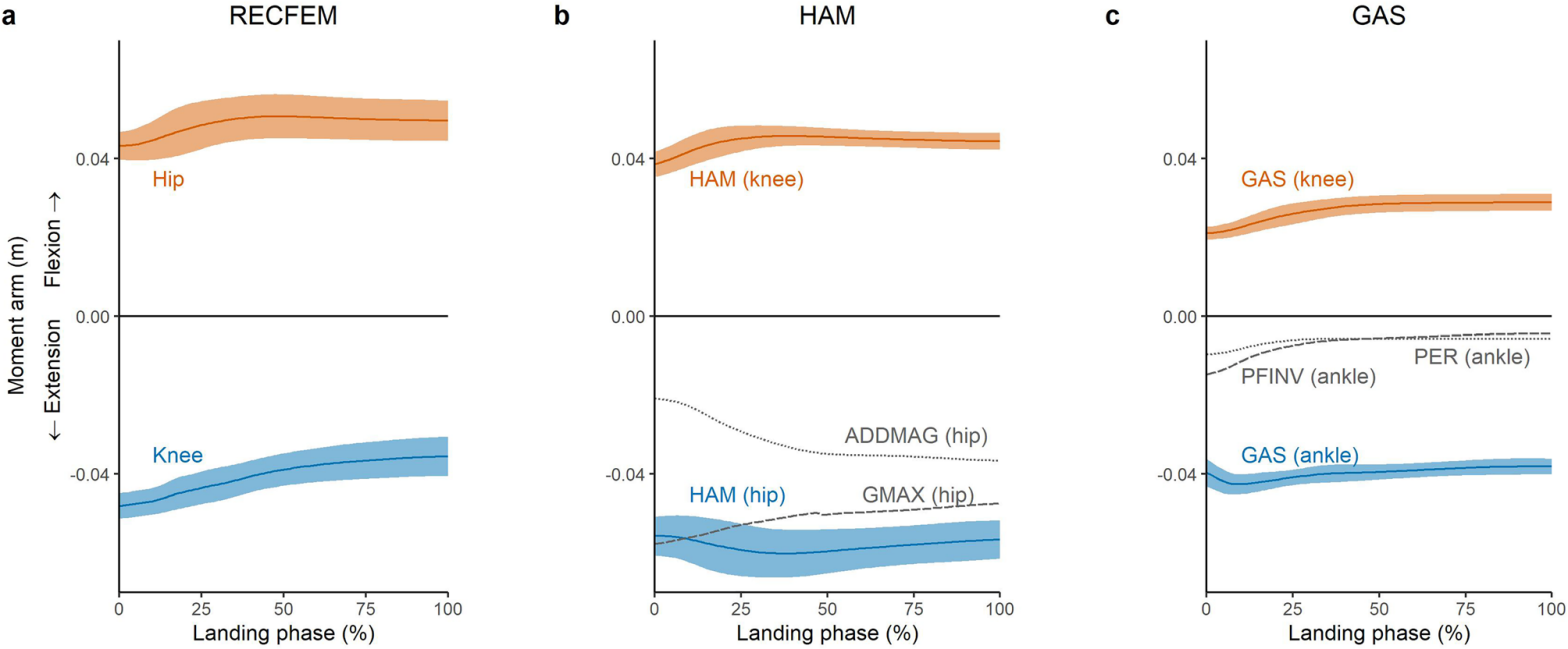
Mean sagittal plane moment arms for biarticular RECFEM (rectus femoris), HAM (hamstrings) and GAS (gastrocnemius) for the landing phase (initial contact to peak knee flexion) of a single-leg drop landing from a 0.31 m height. The blue line and shaded area represent the mean and SD, respectively, of each biarticular muscle’s favourable joint extension moment arm. The orange line and shaded area represent the mean and SD, respectively, of each biarticular muscle’s unfavourable joint flexion moment arm. The grey dotted and dashed lines represent the mean moment arms of other major joint extensors: GMAX, gluteus maximus; ADDMAG, adductor magnus; PFINV, plantar flexor invertors (average of tibialis posterior, flexor hallucis and digitorum longus). The VASTI (vastus lateralis, medialis and intermedius) and soleus moment arms were not visualised due to common attachments with the RECFEM and GAS, respectively.

The relatively lower contributions to joint moments from the bi-articular muscles could be due to several reasons. For example, RECFEM and GAS have comparatively small physiological cross-sectional areas (and thus maximum isometric force-producing capabilities) compared to their uni-articular counterparts, VASTI and SOLEUS, respectively^6^. Another consideration is that bi-articular muscles can produce antagonist action at the other lower limb joints that they span (Figs. 6, 8). For example, HAM may contribute favourably to the production of an internal extension moment at the hip, but this would come at the cost of inducing a counter-productive internal flexion moment at the knee. Hence, relative to GMAX, HAM had a lower recruitment and thus contribution to the hip extension moment, despite having a similar physiological cross sectional area^6^ and greater hip extension moment arm (Fig. 8b). Greater reliance on uni-articular muscles has also been reported for thigh muscles during a bilateral squatting task, where the exercise induced fluid shifts suggested greater recruitment of the uni-articular VASTI and ADDMAG, rather than the bi-articular HAM and RECFEM^44^ to perform simultaneous hip and knee extension. Despite their potentially antagonistic roles, bi-articular muscles may play other critical roles during dynamic tasks such as landing. For example, landing tasks are known to place high demands on the knee joint and are a commonly cited mechanism of anterior cruciate ligament injury^1^. The HAM contributes to knee contact forces (Fig. 7b) and posterior tibial shear forces^29,31^, and may therefore serve a protective role due to their ability to stabilise the knee and unload the anterior cruciate ligament^45^.

### Contribution to frontal plane net joint moments

Due to the single-leg nature of the landing task investigated, the contribution of muscles to the lower limb joint moments in the frontal plane are also of importance. For example, the muscles that contribute to the hip abduction moment may be the key muscles that prevent contralateral pelvic drop and medial knee deviation, which occur when the hip adducts and internally rotates. Our data suggests that the major contributor to the hip abduction moment was GMED, whereas HAM and ADDMAG tended to produce a hip adduction moment (Fig. 6d). These observed roles are similar to previously reported roles in sidestep cutting^28^ and running^43^. At the ankle, the primary contributors to the ankle inversion moment (PFINV and SOLEUS) were similar to those observed during walking and running^43^, and may be important to prevent frontal plane collapse (eversion) of the ankle. The primary contributor to the ankle eversion moment were PER and DORSI. Given that inversion sprains commonly occur during landing tasks^46^, these muscles could be key targets for interventions aiming to limit risk of these injuries. This observation is somewhat supported by a case report^47^, in which authors reported altered recruitment patterns of these muscle groups (as determined via EMG analysis of the peroneus longus and tibialis anterior) in cases of inversion “rolling” of the ankle during drop landing.

### Joint contact loading

To validate our estimates of joint contact loading, we compared our estimates to in-vivo data from running (running being the highest impact task for which in-vivo data are available). Overall, the joint contact forces observed in this work were much higher than in-vivo data from running. For example, in-vivo knee and hip joint contact forces have been reported to be up to ~ 4 to 5.5 BW during running at velocities of ~ 1 to 2.5 m/s^48–51^. The peak knee and hip contact forces observed in the present work were 10.9 BW and 8.3 BW, respectively. Given the task-based differences, and the fact that in-vivo data are only available from individuals with total joint replacement, the substantially larger forces observed in our work were expected and we do not believe these discrepancies invalidate our findings. Whilst in-vivo data for single-leg drop landing are not available, in-silico work (i.e. musculoskeletal modelling) has demonstrated peak knee contact forces of ~ 8.3 to 10.7 BW during single-leg drop landing from a 0.3 m height^42^, providing confidence in our computations.

To our knowledge, no prior studies have computed muscle force contributions to joint contact loading during single-leg landing. However, a growing body of studies have computed muscle force contributions to contact forces at the knee and hip for other locomotion tasks. For example, prior work investigating the stance phase of walking, running and sidestep cutting have shown the quadriceps to be the dominant contributor to knee joint compressive loading^7,29,40,52–55^, similar to our observations (Fig. 7b). Additionally, notable contributions to knee contact loading from the GAS and HAM observed in the present work are also consistent with these prior studies. The present work also suggests that the hip joint contact force is primarily provided by the GMAX and GMED (Fig. 7a). Prior work investigating the stance phase of walking and running have also identified these muscles as the primary contributors to hip contact loading^7,56–58^. However, these studies have typically shown GMED to provide the greatest contribution to hip compressive loading, whereas our work suggest the GMAX is the dominant contributor. These discrepant findings highlight the task-specific nature of muscle function, which is dependent on kinematics of all segments in the system.

Despite the majority of the compressive contact force being due to muscles spanning the joint of interest, other muscles can also contribute substantially to forces at joints they do not span due to dynamic coupling^12^. For example, we found the SOLEUS to contribute to the contact force at the knee (Fig. 7b), an observation consistent with prior studies investigating walking^52,54^ and sidestep cutting^40^. The ability of muscles to contribute to forces at joints they do not span may have important implications for joint injuries and degeneration in which contact forces may be implicated.

### Limitations

Our study involved a convenience sample of recreationally active healthy adult males, thus our conclusions cannot be extrapolated to pathological populations, other age groups or females. Additionally, our participants were not given any specific coaching on their landing technique. The observed variability in some muscles (e.g., SOLEUS and VASTI, Fig. 2) may suggest that different participants naturally adopted different landing techniques, possibly varying between an ankle and knee dominant approach. We did not obtain a sample size large enough to conduct statistical analysis to further explore the features of landing or different landing techniques, but this remains a promising area for future research and may help to inform interventions aiming to improve landing technique (e.g., to reduce injury risk). The task itself was also performed in a well-controlled laboratory environment. Whilst this is an essential first step, future research may aim to investigate similar variables in more “real-world” scenarios, such as performing a landing task whilst reacting to an external stimulus (e.g., catching a ball).

This work required the use of a foot–ground contact model to decompose GRFs into individual muscle contributions. Prior research has shown the various foot–ground contact models can produce different estimates of muscle function for certain muscles, in certain planes^39^, and it is currently unclear which model produces the most accurate results. The universal “rolling on ground” constraint was used for the present work since it adequately described movement of the foot relative to the ground in the chosen task (in a qualitative sense), reproduced experimental GRFs (Supplementary Fig. S3 online), and has been used in prior work^21,22,26,59^.

Muscle forces estimated in the present work cannot be directly validated, as in-vivo muscle forces are not practically feasible to measure^7^. However, the EMG-assisted methodology employed has been shown to be capable of accurately predicting in-vivo joint contact forces^60^, which serves as an indirect validation of muscle forces due to the high dependency of joint contact forces on muscle forces^7^. Furthermore, the EMG-assisted methodology produced good agreement between experimentally recorded EMG and predicted excitations (Supplementary Fig. S2 online), and thus helped to ensure that time-varying trends in our predicted muscle forces were physiologically plausible and participant specific.

## Conclusion

In conclusion, this study quantified lower limb muscle function during a single-leg drop landing task. We found that the uni-articular joint extensors (VASTI, SOLEUS and GMAX) produced the greatest muscle forces and negative mechanical work, predominately at the beginning of the landing phase. These same muscles provided the greatest resistance to flexion of the lower limb joints, along with contributions from the bi-articular HAM (at the hip), RECFEM (at the knee) and GAS (at the ankle). Frontal plane collapse was resisted by the GMED (at the hip) and the SOLEUS and PFINV (at the subtalar joint). Downward momentum of the centre-of-mass was primarily arrested by the SOLEUS, VASTI, GAS, RECFEM and GMAX, whilst the quadriceps (VASTI and RECFEM) were primarily responsible for arresting forward centre-of-mass momentum upon landing. Muscles were also found to contribute substantially to joint contact loading, in some cases including joints that they do not span. This work adds to a growing body of knowledge on muscle function during dynamic tasks and may have important implications for athletic training by indicating which muscles may serve as targets for interventions aiming to improve landing performance.

## Supplementary Information

Supplementary Information.

## Data Availability

Non-identifiable datasets generated during and/or analysed during the current study are available from the corresponding author on reasonable request.
