# Supplementary material for: Muscle function during single leg landing
Source: Sci Rep. 2022 Jul 7;12:11486. doi: 10.1038/s41598-022-15024-w (PMC9262956; doi:10.1038/s41598-022-15024-w)
Supplement: Supplementary file 1 — Supplementary Information. [file 41598_2022_15024_MOESM1_ESM.pdf]

Supplementary material

The following document contains supplementary data for the validation of our simulations for the following manuscript:

Muscle function during single leg landing

Authors

Nirav Maniar[1,2], Anthony G Schache[3], Claudio Pizzolato[4,5], David A Opar[1,2]

[1]School of Behavioural and Health, Australian Catholic University, Fitzroy, Victoria, Australia

[2]Sports Performance, Recovery, Injury and New Technologies (SPRINT) Research Centre, Australian Catholic University, Fitzroy, Victoria, Australia

[3]La Trobe Sport and Exercise Medicine Research Centre, La Trobe University, Bundoora, Victoria, Australia

[4]Griffith Centre of Biomedical and Rehabilitation Engineering, Menzies Health Institute Queensland, Griffith University, Gold Coast, Queensland, Australia

[5]School of Health Sciences and Social Work, Griffith University, Gold Coast, Queensland, Australia.

The data below includes the comparison of simulated and experimental joint moments, comparison of simulated and experimental excitations, comparison of simulated and experimental ground reaction forces, comparison of joint reaction forces computed via simulated and experimental ground reaction forces, and a table outlining the synthesis methods of muscle force estimation.

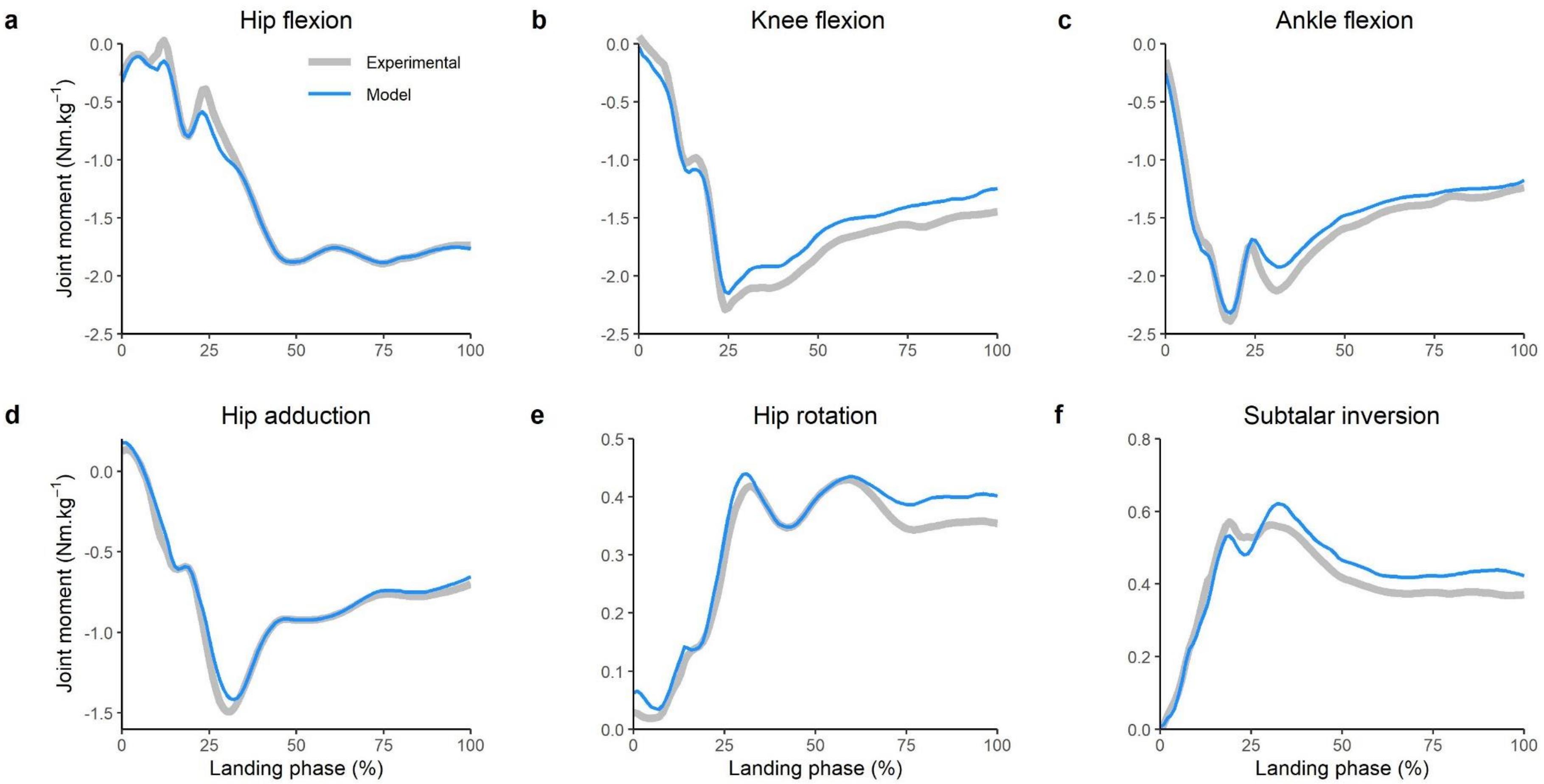

Supplementary Figure S1. Comparison of model (blue) and experimental (grey, from inverse dynamics) body mass normalised joint moments for the landing phase (initial contact to peak knee flexion) of a single-leg drop landing task from a 0.31m height. Positive values are indicated by the title of each subplot.

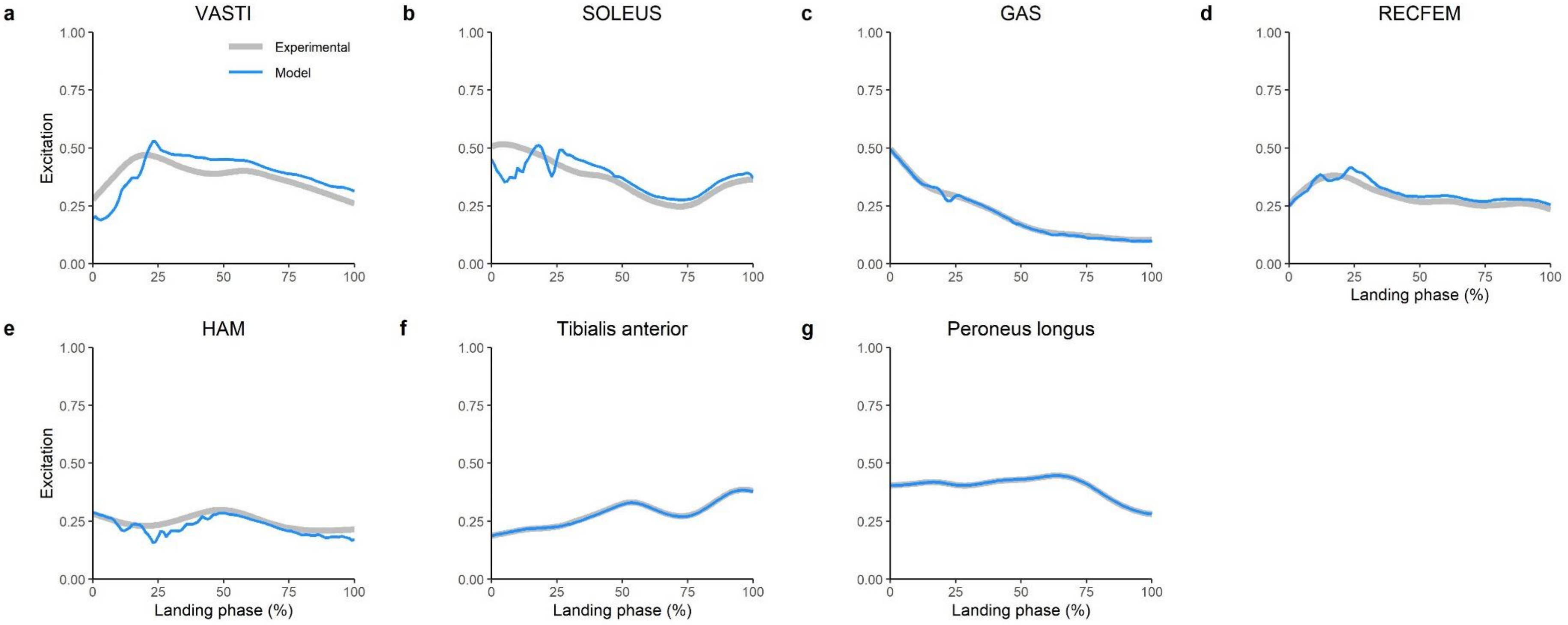

Supplementary Figure S2. Comparison of model (blue) and experimental (grey, from surface electromyography) excitations for the landing phase (initial contact to peak knee flexion) of a single-leg drop landing task from a 0.31m height. VASTI, average of vastus lateralis and medialis signals, SOLEUS, soleus, GAS, average of lateral and medial gastrocnemius signals, RECFEM, rectus femoris, HAM, average of medial and lateral hamstring signals.

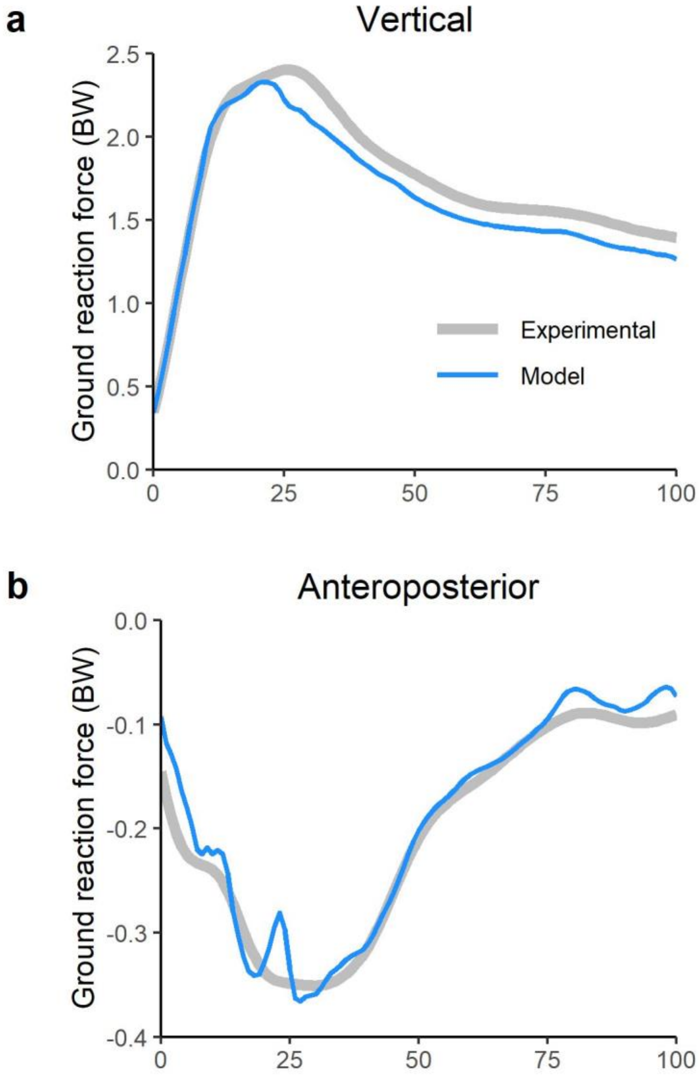

Supplementary Figure S3. Comparison of model (blue, from "rolling on ground" constraint) and experimental (grey, from force plate) bodyweight (BW) normalised ground reaction forces for the landing phase (initial contact to peak knee flexion) of a single-leg drop landing task from a 0.31m height. Positive values indicate superior (panel a) and anterior (panel b) directed ground reaction forces.

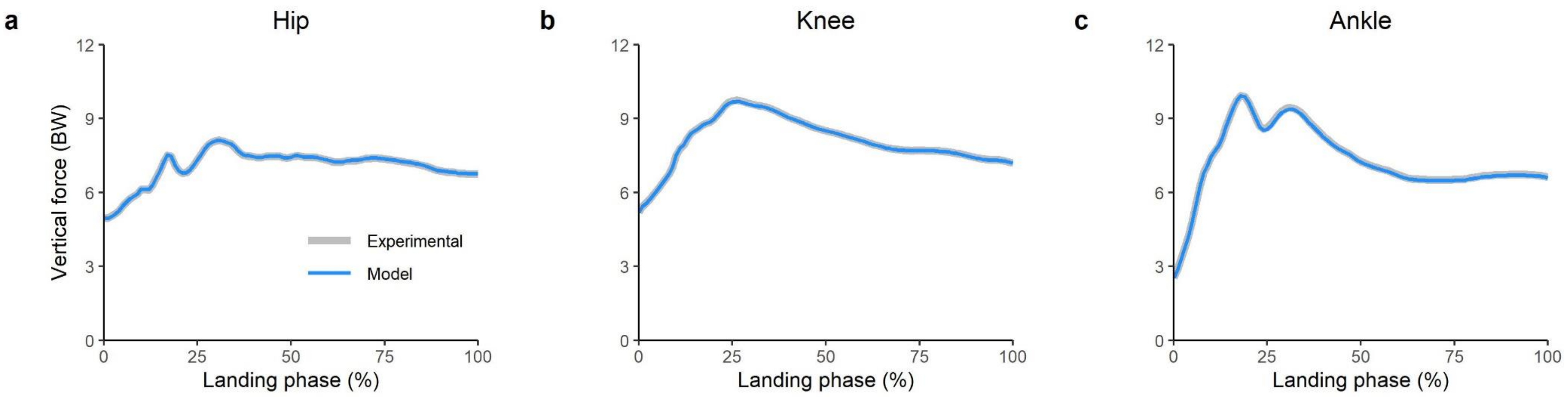

Supplementary Figure S4. Comparison of model (blue, from "rolling on ground" constraint derived ground reaction forces) and experimental (grey, from force plate derived ground reaction forces) bodyweight (BW) normalised joint contact force for the landing phase (initial contact to peak knee flexion) of a single-leg drop landing task from a 0.31m height. Positive values represent a compressive force.

Supplementary Table 1. Mapping of lower-limb muscles, corresponding model actuators and excitation inputs.

Abbreviation	Muscle group	Muscles	Model actuator	Excitation input
ADDLONG	Adductor longus/brevis	Adductor brevis	addbrev	Synthesised
		Adductor longus	addlong	Synthesised
ADDMAG	Adductor magnus	Adductor magnus	addmagDist	Synthesised
			addmagIsch	Synthesised
			addmagMid	Synthesised
			addmagProx	Synthesised
BFSH	Biceps femoris short head	Biceps femoris short head	bfsh	Synthesised
DORSI	Dorsiflexors	Extensor digitorum longus	edl	Synthesised
		Extensor hallucis longus	ehl	Synthesised
		Tibialis anterior	tibant	EMG (Tibialis anterior)
GAS	Gastrocnemius	Lateral gastrocnemius	gaslat	EMG (Lateral gastrocnemius)
		Medial gastrocnemius	gasmed	EMG (Medial gastrocnemius)
GMAX	Gluteus maximus	Gluteus maximus	glmax1	Synthesised
			glmax2	Synthesised

			glmax3	Synthesised
GMED	Gluteus medius/minimus	Gluteus medius	glmed1	Synthesised
			glmed2	Synthesised
			glmed3	Synthesised
		Gluteus minimus	glmin1	Synthesised
			glmin2	Synthesised
			glmin3	Synthesised
HAM	Biarticular hamstrings	Biceps femoris long head	bflh	EMG (Lateral hamstring)
		Semimembranosus	semimem	EMG (Medial hamstring)
		Semitendinosus	semiten	EMG (Medial hamstring)
ILPSO	Iliopsoas	Iliacus	iliacus	Synthesised
		Psoas major	psoas	Synthesised
PER	Peroneals	Peroneus brevis	perbrev	EMG (Peroneus longus)
		Peroneus longus	perlong	EMG (Peroneus longus)
PFINV	Plantar-flexor-invertors	Flexor digitorum longus	fdl	Synthesised
		Flexor hallucis longus	fhl	Synthesised

		Tibialis posterior	tibpost	Synthesised
PIRI	Piriformis	Piriformis	piri	Synthesised
RECFEM	Rectus femoris	Rectus femoris	recfem	EMG (Rectus femoris)
SOLEUS	Soleus	Soleus	soleus	EMG (Soleus)
VASTI	Vasti	Vastus intermedius	vasint	EMG (Vastus lateralis)
		Vastus lateralis	vaslat	EMG (Vastus lateralis)
		Vastus medialis	vasmed	EMG (Vastus medialis)

EMG, excitations were derived from collected surface electromyography data; Synthesised, excitations were derived from a static optimisation algorithm.
